# Outcomes at 6 months are related to brain structural and white matter microstructural reorganization in idiopathic tinnitus patients treated with sound therapy

**DOI:** 10.1002/hbm.25260

**Published:** 2020-10-24

**Authors:** Qian Chen, Han Lv, Zhaodi Wang, Xuan Wei, Pengfei Zhao, Zhenghan Yang, Shusheng Gong, Zhenchang Wang

**Affiliations:** ^1^ Department of Radiology Beijing Friendship Hospital, Capital Medical University Beijing People's Republic of China; ^2^ Department of Otolaryngology Head and Neck Surgery Beijing Friendship Hospital, Capital Medical University Beijing People's Republic of China

**Keywords:** brain reorganization, idiopathic tinnitus, sound therapy, tract‐based spatial statistics, voxel‐based morphometry

## Abstract

This study aimed to explore brain structural and white matter microstructural reorganization in the early stage of tinnitus and identify brain alterations that contribute to its relief after 6 months of sound therapy. We studied 64 patients with idiopathic tinnitus, including 29 patients who were categorized into an effective group (EG) and 35 who were categorized into an ineffective group (IG) according to the 6‐month follow‐up improvement of the Tinnitus Handicap Inventory score, along with 63 healthy controls (HCs). All participants underwent structural and diffusion tensor imaging scanning on a 3‐T magnetic resonance system. Differences in brain gray/white matter volume and white matter microstructure were evaluated using voxel‐based morphometry analysis and tract‐based spatial statistics among the three groups. Associations between brain reorganization and the improvement of tinnitus symptoms were also investigated. Compared with EG patients, IG patients experienced a significant gray matter volume decrease in the right middle frontal gyrus (MFG)/right precentral gyrus (PreCG). Meanwhile, both EG and IG patients showed significant changes (decrease or increase) in brain white matter integrity in the auditory‐related or nonauditory‐related white matter fiber tracts compared with HCs, while EG patients showed decreased axial diffusivity in the bilateral middle cerebellar peduncle (MCP) compared with IG patients. We combined the gray matter change of the MFG/PreCG and the white matter integrity of the bilateral MCP as an imaging indicator to evaluate the patient's prognosis and screen patients before treatment; this approach reached a sensitivity of 77.1% and a specificity of 82.8%. Our study suggests that there was a close relationship between brain reorganization and tinnitus improvement. The right MFG/PreCG and bilateral MCP may be indicators that can be used to predict prognoses in patients with idiopathic tinnitus and may be used to screen patients before sound therapy. These findings may provide new useful information that can lead to a better understanding of the tinnitus mechanism.

## Graphical abstract

We found that effective group and ineffective group patients showed distinct brain reorganization patterns. We observed that associations exist between the brain reorganizations and the improvement of tinnitus. The brain reorganizations may be indicators to predict prognosis and screen patients before sound therapy.

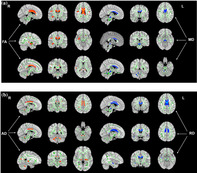

**Abbreviations**
AD: axial diffusivity
CC: corpus callosum
CSF: cerebrospinal fluid
DTI: diffusion tensor imaging
EG: effective group
FA: fractional anisotropy
GMV: gray matter volume
HC: healthy control
IG: ineffective group
MCP: middle cerebellar peduncle
MD: mean diffusivity
MFG: middle frontal gyrus
MNI: Montreal Neurological Institute
PreCG: precentral gyrus
RD: radial diffusivity
TBSS: tract‐based spatial statistics
THI: tinnitus handicap inventory
VBM: voxel‐based morphometry
WM: white matter
WMV: white matter volume

## 1 INTRODUCTION

As an auditory hallucination without external sound stimulation, tinnitus and its related or secondary abnormalities (such as distress, depression, and anxiety) seriously affect the quality of patients' lives (Bhatt, Lin, & Bhattacharyya, 2016; Sereda, Xia, el Refaie, Hall, & Hoare, 2018). Previous studies have shown that tinnitus is a problem of the central nervous system (CNS) (Eggermont & Roberts, 2004), and it can cause significant remodeling of the brain structure and function, which is closely related to patients' clinical performance or may even be the main factor responsible for tinnitus (Chen et al., 2015; Chen et al., 2020; Han, Na, et al., 2019; Han, Yawen, et al., 2019; Lv et al., 2020; Ryu, Park, Byun, Jahng, & Park, 2016; Schmidt, Zimmerman, Bido Medina, Carpenter‐Thompson, & Husain, 2018). However, due to the complexity of the tinnitus mechanism (Adjamian, Hall, Palmer, Allan, & Langers, 2014; de Ridder et al., 2014; Vanneste & de Ridder, 2012) and the limitations of neuroimaging research methods, it is unclear which exact brain regions or neural pathways (Schaette & McAlpine, 2011; Vanneste, Alsalman, & de Ridder, 2019) play an important role in the occurrence and development of tinnitus. Thus, current treatments cannot achieve ideal therapeutic effects for each individual patient (Langguth, Kreuzer, Kleinjung, & de Ridder, 2013). Effective precision therapeutic strategies that target the mechanism of tinnitus (such as specific brain areas) are still urgently needed.

To date, many therapeutic interventions have been applied to the treatment of idiopathic tinnitus, such as drug therapy, cognitive behavioral therapy, cochlear implants, retraining therapy, transcranial magnetic stimulation, sound therapy, and even acupuncture (Langguth et al., 2013; Zenner et al., 2017). In the abovementioned treatment methods, sound therapy, such as frequency unmodulated noise generators involving the use of recorded noise, a special tinnitus noiser, or a tinnitus masker device (Jastreboff, 1999; Oishi et al., 2013), has been widely suggested in the treatment of idiopathic tinnitus in many studies (Han, Na, et al., 2019; Han, Yawen, et al., 2019; Jastreboff, 1999; Lv et al., 2020; Oishi et al., 2013). Meanwhile, sound therapy has been listed as an option in clinical practice guidelines (Henry, Schechter, Nagler, & Fausti, 2002; Tunkel et al., 2014) and even as a first line of management for tinnitus patients (together with other hearing aids, information, and advice) (Hoare, Edmondson‐Jones, Sereda, Akeroyd, & Hall, 2014; Hobson, Chisholm, & El, 2012; Sereda, Hoare, Nicholson, Smith, & Hall, 2015; Tutaj, Hoare, & Sereda, 2018).

There have been a few noninvasive studies on the effect of sound therapy in patients with idiopathic tinnitus so far, but they have reported divergent results (Han, Na, et al., 2019; Han, Yawen, et al., 2019; Lv et al., 2020; Moffat et al., 2009; Oishi et al., 2013; Parazzini, del Bo, Jastreboff, Tognola, & Ravazzani, 2011). For example, some researchers using neuroimaging methods have found that sound therapy has a normalizing effect on the enhanced or increased functional connectivity in some regions that are related to tinnitus; this normalizing effect may represent less involvement of the noise‐canceling system (Han, Na, et al., 2019; Han, Yawen, et al., 2019; Lv et al., 2020). Furthermore, these studies also showed that after this treatment, clinical performance of patients had also been significantly improved when using the Tinnitus Handicap Inventory (THI) changes as the evaluation standard, and the brain alterations of patients were closely related to clinical performance (Han, Na, et al., 2019; Han, Yawen, et al., 2019; Lv et al., 2020). However, some studies show that sound therapy has no measurable improvement in the treatment of tinnitus (Moffat et al., 2009; Oishi et al., 2013; Parazzini et al., 2011), which may be due to the low methodological quality and insufficient effect size that was revealed via statistical analysis. Thus, the mechanism that results in effective or ineffective sound therapy outcomes for tinnitus is still unclear, and it cannot be clarified whether the treatment method needs to be improved or whether the treatment is not suitable for some patients. Additionally, screening patients with idiopathic tinnitus for more effective treatment before applying sound therapy is still a considerable challenge.

Accordingly, to address these issues, in the present study, we investigated the long‐term (6 months) prognosis of performing sound therapy for idiopathic tinnitus patients. Moreover, we explored brain structural alterations using voxel‐based morphometry (VBM) analysis and tract‐based spatial statistics (TBSS) and studied the relationship between brain changes and prognosis. According to the follow‐up THI scores, the patients were divided into two groups: an effective group (EG) and an ineffective group (IG). By comparing the differences in brain alterations between the two groups after sound therapy, we aimed to reveal the possible different mechanisms of tinnitus in patients with different therapeutic effects to guide the selection of optimal treatment methods for patients with idiopathic tinnitus prior to treatment.

## 2 MATERIALS AND METHODS

### 2.1 Participants

Sixty‐four untreated persistent idiopathic tinnitus patients (right‐handed) were enrolled in this study. Sixty‐four age‐, sex‐, and education‐matched right‐handed healthy volunteers were also recruited as healthy controls (HCs). However, one healthy volunteer was excluded due to the relatively long data acquisition time (as he could not refrain from moving his body for a long time); thus, 64 patients and only 63 HCs were included in our study. The 64 selected patients fit the following inclusion criteria for the present study: persistent idiopathic tinnitus (≥6 months persistently and ≤48 months), availability of high‐quality brain structural and diffusion tensor imaging (DTI) data, no history of associated brain diseases confirmed by conventional MRI, and no preexisting mental illness or cognitive disorder affecting the brain alteration outcome. Tinnitus was present as a single high/low‐pitched sound and/or two high/low‐pitched sounds without any rhythm. Based on audiogram results, a proportion of patients did not have significant hearing loss, which was defined as more than 25 dB hearing loss at frequencies ranging from 250 to 8 kHz in pure tone audiometry examination, while others had different degrees of tinnitus‐related hearing loss. Patients with the following conditions were excluded from this study: pulsatile tinnitus, hyperacusis on physical examination, otosclerosis, sudden deafness, Ménière's disease, and other neurological diseases. We asked all the patients to complete the THI (Newman et al., 1996) and visual analog scale (VAS) at the time of admission to assess their disease severity. We also evaluated the current severity of depression and distress. The hearing condition of HCs was evaluated by audiologists using a questionnaire, and no HCs had tinnitus in the last year. Other exclusion criteria for HCs were the same as patients described above.

### 2.2 Sound therapy

To characterize the tinnitus type and prepare for treatment, all tinnitus patients were evaluated for four main factors: tinnitus loudness matching, pitch matching, minimum masking level, and residual inhibition. After this evaluation, narrow band noise was applied for 20 min three times per day for 6 months to treat tinnitus. The loudness of the sound used to treat each tinnitus patient was set as tinnitus loudness plus 5 dB. The frequency was set as a 1 kHz narrow band when setting the tinnitus frequency as the middle point of the delivered sound (tinnitus frequency ± 0.5 kHz). During the process, all the patients were asked to complete the THI to assess the tinnitus severity before and after treatment, and the primary outcome of this study was the THI score change. Consistent with prior research, we defined tinnitus patients with a THI score reduced to 16 points or a reduction of 17 points or more as the EG (Zeman et al., 2011), while those with a decrease in THI scores that did not reach these two conditions were defined as IG. Tinnitus patients were divided into two groups; 29 were classified into an EG and 35 were classified into an IG according to the outcome after the treatment. HCs were not subjected to any kinds of sound during the study. We also calculated the ^Δ^THI score and the % improvement of the THI score in all patients with tinnitus, which were defined as follows: ^Δ^THI score = THI_baseline_ − THI_treated_, % improvement of the THI score = (THI score at 6 months follow‐up‐THI score on admission)/THI score on admission × 100%.

The present study was approved by the Institutional Review Board of Beijing Friendship Hospital, Capital Medical University, Beijing, China. Written informed consent was obtained from all study subjects after a full explanation of the procedure involved. The research was completed in accordance with the Declaration of Helsinki. The registration number of our study on ClinicalTrials.gov is NCT03764826.

### 2.3 Image acquisition

All images were obtained using a 3.0 T MRI system (Prisma, Siemens, Erlangen, Germany) with a 64‐channel phase‐array head coil. To exclude any possible abnormality in the brain, we scanned a conventional brain axial T2 sequence first. High‐resolution three‐dimensional (3D) structural T1‐weighted images were acquired using a 3D magnetization‐prepared rapid gradient‐echo sequence (MP‐RAGE) with the following parameters: repetition time (TR) = 2,530 ms; echo time (TE) = 2.98 ms; inversion time (TI) = 1,100 ms; flip angle (FA) = 7°; number of slices = 192; slice thickness = 1 mm; bandwidth = 240 Hz/Px; field of view (FOV) = 256 × 256 mm^2^; and matrix = 256 × 256, resulting in an isotropic voxel size of 1 × 1 × 1 mm^3^. The DTI experiments were performed using a single‐shot gradient‐echo echo‐planar imaging sequence with the following imaging parameters: TR = 8,500 ms, TE = 63 ms, matrix = 128 × 128, acquisition voxel size 2 × 2 × 2 mm^3^, FOV = 224 × 224 mm^2^, nonzero *b* value = 1,000 s/mm^2^, gradient directions = 64, slice thickness = 2 mm, and bandwidth = 2,232 Hz/Px. A total of 74 contiguous slices parallel to the anterior commissure‐posterior commissure line were acquired.

During the scanning process, we used tight but comfortable foam padding and earplugs to minimize head motion and reduce imaging noise, respectively. The participants were asked to close their eyes, stay awake, breathe evenly, and try to avoid specific thoughts.

### 2.4 Image analysis

#### 2.4.1 Structural data processing and VBM analysis

We used the CAT12 package (http://www.neuro.uni-jena.de) implemented in Statistical Parametric Mapping (SPM) software (version 12; https://www.fil.ion.ucl.ac.uk/spm) for postprocessing structural data. SPM12 was installed in MATLAB 2016a (Math Works, Natick, MA). First, all the structural images were checked by two experienced radiologists to ensure no apparent artifacts caused by factors such as head motion, susceptibility artifacts, and instrument malfunction. Then, the images were manually reoriented to place the anterior commissure at the origin and the anterior–posterior commissure in the horizontal plane if necessary. Next, the structural images were segmented into gray matter, white matter (WM) and cerebrospinal fluid areas using the unified standard segmentation option in SPM12. The individual gray matter and WM components were then normalized into standard Montreal Neurological Institute (MNI) space using the Diffeomorphic Anatomical Registration through Exponentiated Lie Algebra (DARTEL) algorithm (Ashburner, 2007) after segmentation. The normalized gray matter and WM components were modulated to generate the relative gray matter volume (GMV) and WM volume (WMV) by multiplying by the nonlinear part of the deformation field at the DARTEL step. The Gaussian kernel used to smooth the GMV/WMV images was 6‐mm full‐width at half‐maximum.

#### 2.4.2 Data processing and DTI

We used the FSL 5.0.3 software package (Centre for FMRIB, Oxford University, Oxford, UK; https://fsl.fmrib.ox.ac.uk/fsl/fslwiki/) (Smith et al., 2006) for DTI data postprocessing. The postprocessing main steps were as follows: (a) all the DTI images were visually checked to eliminate images with apparent artifacts and (b) eddy current corrections were applied, and motion artifacts were removed using affine alignment. Next, nonbrain tissues were removed using the brain extract tool. This process reduces the computation times of the DTI fitting and tracking processes and improves the accuracy of the spatial registration. After that, the diffusion tensor of each voxel was fitted using a linear least squares algorithm, and the fractional anisotropy (FA), mean diffusivity (MD), axial diffusivity (AD), and radial diffusivity (RD) maps were calculated based on the eigenvalues of diffusion tensors (Raya et al., 2012). For TBSS analysis, the main procedures were as follows: the entire FA dataset was nonlinearly coregistered to the MNI FA template in the FSL database. Next, a mean FA skeleton from the mean FA images of all of the subjects was derived; this mean FA skeleton represented the center of the WM tracts common to the group. An FA threshold of 0.2 was used to involve only the major WM pathways while eliminating peripheral tracts that are susceptible to misregistration. Finally, each aligned FA map was projected back onto the skeleton to generate a subject‐specific FA skeleton. The processes of nonlinear warping and skeleton projection of the FA maps were also applied to MD, AD, and RD maps.

### 2.5 Statistical analyses

For T1 MP‐RAGE images, using the general linear model (GLM) in SPM12, a voxelwise one‐way analysis of variance followed by post hoc analyses was applied to compare the GMV and WMV differences in the whole brain among the EG, IG and HC group (voxel‐level uncorrected *p* < .001, nonstationary cluster‐level FWE correction with *p* < .05), and age and sex served as nuisance covariates. For DTI data, TBSS analysis using a nonparametric permutation test (5,000 permutations) was performed to compare the differences in the four DTI indices among the three groups. The permutation test was performed with a fixed‐effect GLM with age and sex as nuisance covariates. Statistical significance was set at *p* < .05 and corrected for multiple comparisons using the threshold‐free cluster enhancement method. Next, the regions that exhibited significant alterations in DTI indices among the three groups were defined as the regions of interest (ROIs), and the mean values of each ROI of each subject were extracted for subsequent post hoc analyses using SPSS Statistics software, version 23.0 (IBM Inc., Armonk, NY). Moreover, the mean GMV and WMV of each cluster with statistical significance were also extracted for subsequent analyses. Finally, partial correlation analysis was performed to explore any potential associations between the patients' clinical variables (such as the duration, THI scores, and VAS scores) and the brain changes (GMV, WMV, and four DTI indices) after removing any age and sex effects. The last several steps were performed using SPSS as well. All data in this study were assessed by the Kolmogorov–Smirnov test for normality. Data identified as not normally distributed were analyzed using nonparametric tests.

The results of the GMV/WMV and WM microstructural changes were presented using MRIcron (https://www.nitrc.org/projects/mricron) and FSLeyes (https://fsl.fmrib.ox.ac.uk/fsl/fslwiki/FSLeyes).

## 3 RESULTS

### 3.1 Sample characteristics and clinical measurements

Our sample consisted of 64 tinnitus patients with persistent idiopathic tinnitus (29 in the EG and 35 in the IG according to the outcome after treatment) and age‐, sex‐, education‐, and handedness‐matched 63 healthy volunteers. Among the three groups, we did not observe any significant differences in age, sex, handedness, disease duration, or type and laterality of tinnitus. We also obtained the THI scores before and after sound therapy, the changes in THI scores, and the improvement of THI scores. As with % improvement of THI scores, the EG showed significantly greater improvement than the IG (53.17% vs. −15.81%) (Table 1).

**TABLE 1:** Demographic and clinical characteristics of the tinnitus patients and healthy volunteers

Demographic	Tinnitus‐EG (baseline, *n* = 29)	Tinnitus‐EG (treated, *n* = 29)	Tinnitus‐IG (baseline, *n* = 35)	Tinnitus‐IG (treated, *n* = 35)	Controls (*n* = 63)	*p*‐Value
Age, years	48.83 (±12.64)		47.46 (±12.33)		47.05 (±12.19)	.812^a^
Gender	12 males, 17 females		20 males, 15 females		33 males, 30 females	.438^b^
Handedness	29 right‐handed		35 right‐handed		63 right‐handed	>0.99^a^
THI score	69.24 (±21.44)	33.03 (±20.00)	42.74 (±18.71)	47.74 (±19.43)	NA	<.001^c^
^Δ^THI score	36.21 (±18.57)		−4.80 (±10.85)		NA	NA
% Improvement of THI score	53.17% (±19.56%)		−15.81% (±41.09%)		NA	
Duration, months	≥6 and ≤48		≥6 and ≤48		NA	NA
Type^d^	16:2:1:1:6:3		21:6:4:1:2:1		NA	.804^a^
Tinnitus pitch	250–8,000 Hz		250–8,000 Hz		NA	NA
Laterality	4 right, 11 left, 13 bilateral		6 right, 9 left, 20 bilateral		NA	.322^e^

Abbreviations: ANOVA, analysis of variance; EG, effective group; IG, ineffective group; NA, not applicable; THI, Tinnitus Handicap Inventory, ^△^THI score, THI_baseline_ − THI_treated_.

^a^ One‐way ANOVA.

^b^ Chi‐square test.

^c^ Two‐way ANOVA.

^d^ One single high‐pitched sound versus one single low‐pitched sound versus two or more high pitched sounds versus two low pitched sounds versus two or more irregular sounds versus indistinguishable.

^e^ Mann–Whitney *U* test.

### 3.2 Brain structural alterations among the EG, IG, and HC group

**FIGURE 1:**
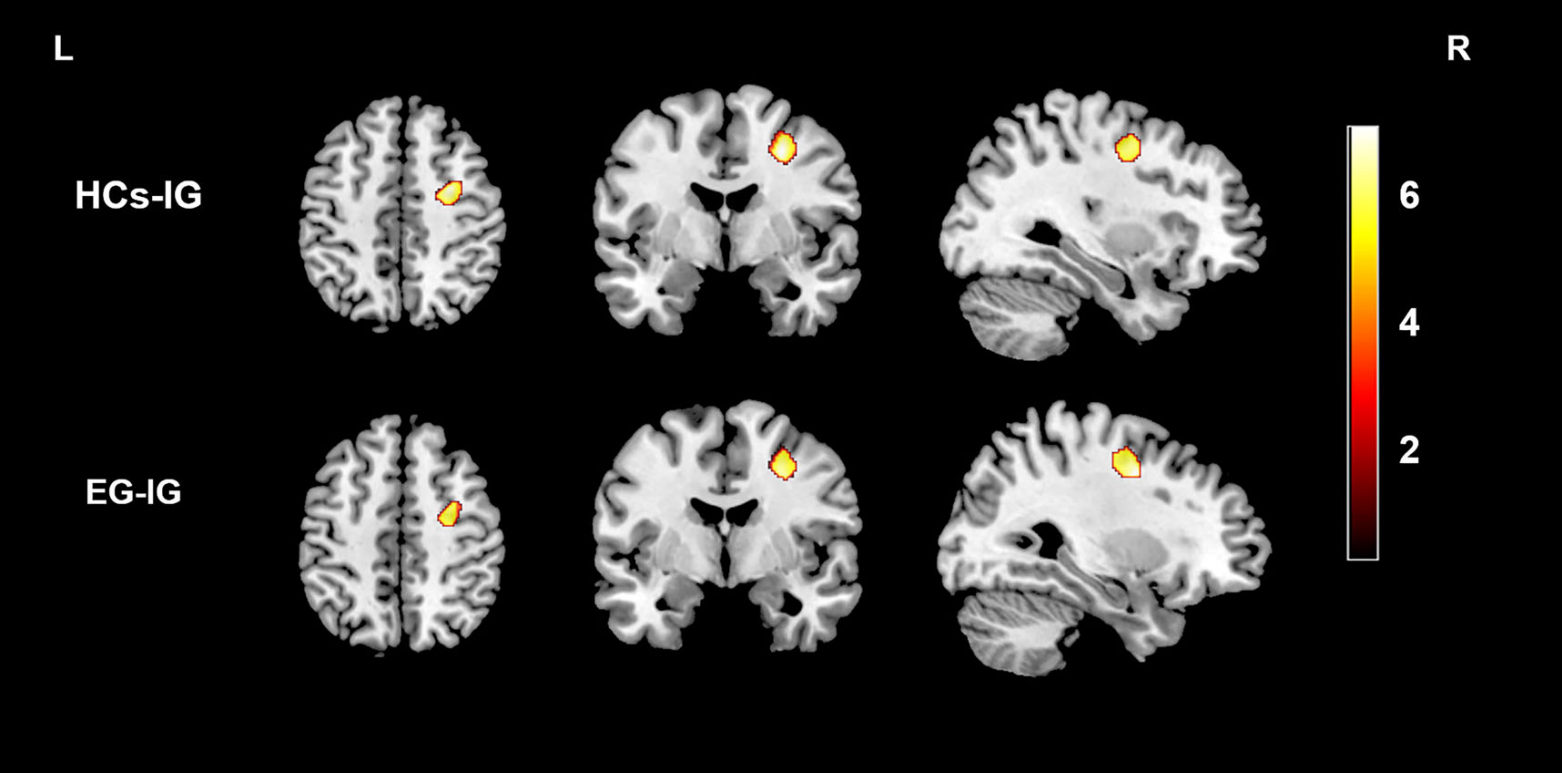
Regions showing differences in GMV between the HC group and IG and between the EG and IG. Compared with participants in the HC group, IG patients exhibited decreased GMV in the right MFG/PreCG. Direct comparison between the EG and IG showed that GMV was reduced in the right MFG/PreCG in IG patients (corrected at the cluster level with FWE *p* < .05). EG, effective group; FWE, familywise error; GMV, gray matter volume; HCs, healthy controls; IG, ineffective group; MFG, middle frontal gyrus; PreCG, precentral gyrus

One‐way analysis of covariance showed two brain regions that had significant differences in GMV, namely, the right middle frontal gyrus (MFG) and right precentral gyrus (PreCG). Compared with the HC group, the IG had significantly decreased GMV in these two regions. A direct comparison between the two patient groups also revealed significantly decreased GMV in the two areas in the IG compared with the EG (Figure 1, Table 2). Moreover, we found that the mean GMV value in the right MFG/PreCG was negatively correlated with the ^Δ^THI score and the % improvement of THI score in all tinnitus patients (*r* = −.286, *p* = .024 and *r* = −.362, *p* = .004, respectively) (Figure 4a,b). However, we did not find any significant differences in WMV among the three groups.

**TABLE 2:** Difference in gray matter volume among EG, IG, and HCs (FWE cluster *p* < .05)

Anatomical region	MNI coordinate			Voxel size	Peak *T* value
	x	y	z		
IG < HCs					
MFG/Precentral_R (aal)	27	−3	44	398	5.36
IG < EG					
MFG/Precentral_R (aal)	29	0	42	353	4.55

Abbreviations: EG, effective group; FWE, family wise error; IG, ineffective group; HCs, healthy controls; MFG, middle frontal gyrus; MNI, Montreal Neurological Institute.

### 3.3 Brain WM microstructural alterations among the EG, IG, and HC group

**FIGURE 2:**
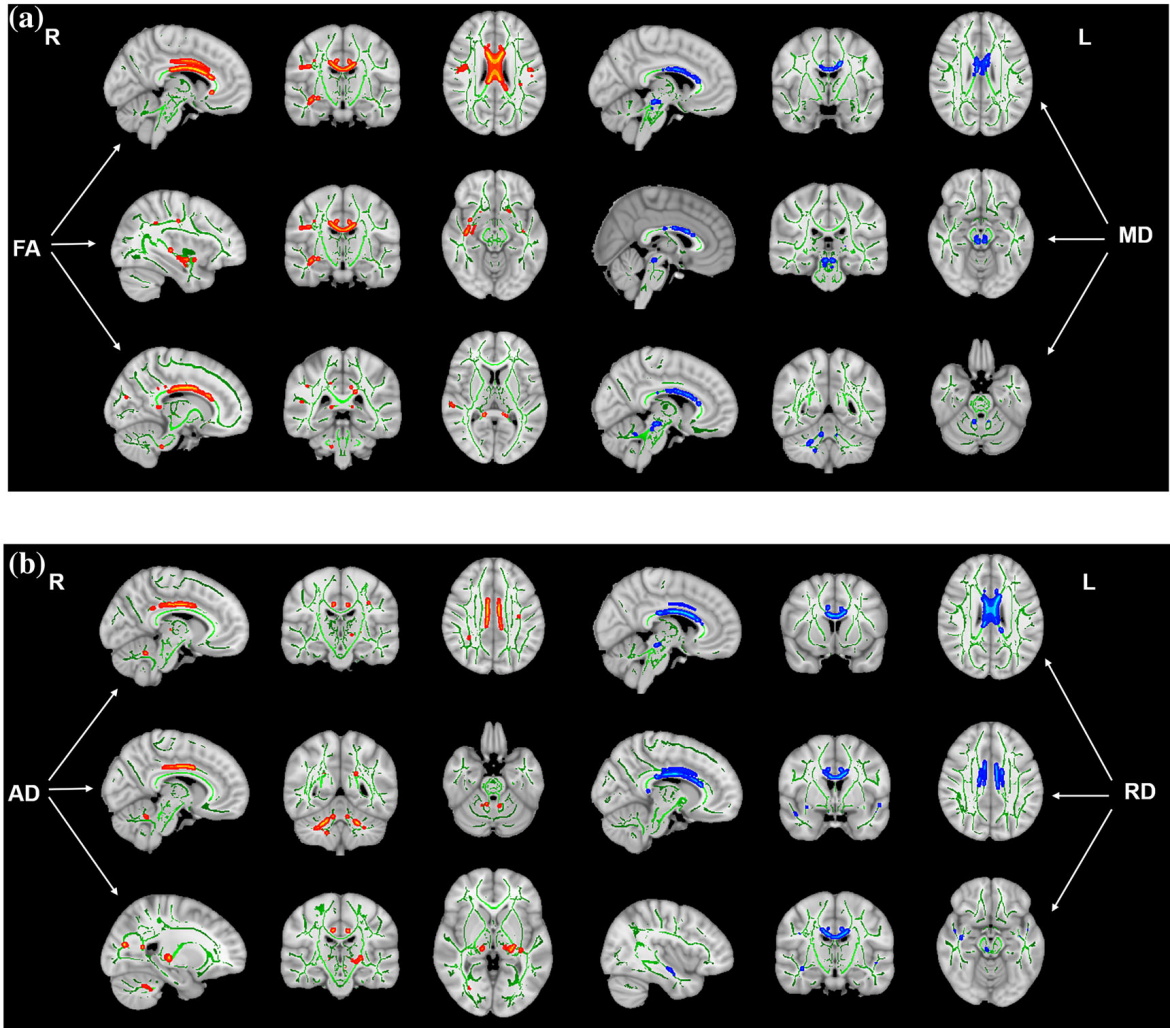
Brain white matter (WM) microstructural changes in patients with idiopathic tinnitus compared with HCs (a) Changed FA and MD values in tinnitus patients compared with HCs. Patients showed significant decreases in FA in the genu and splenium of CC, bilateral cingulum (cingulate gyrus), RILF and RIFOF, RSLF, and RSTG and an increase in MD in the body and genu of the CC and bilateral brainstem. (b) Changes in AD and RD values in tinnitus patients compared with HCs. Patients showed decreased AD in the bilateral cingulum (cingulate gyrus), bilateral MCP, left thalamus, RSLF, and left retrolenticular part of the IC (p < .05, voxel‐level FWE‐corrected). AD, axial diffusivity; CC, corpus callosum; FA, fractional anisotropy; HCs, healthy controls; IC, internal capsule; MCP, middle cerebellar peduncle; MD, mean diffusivity; RD, radial diffusivity; RIFOF, right inferior fronto‐occipital fasciculus; RILF, right inferior longitudinal fasciculus; RSLF, right superior longitudinal fasciculus; RSTG, right superior temporal gyrus

**FIGURE 3:**
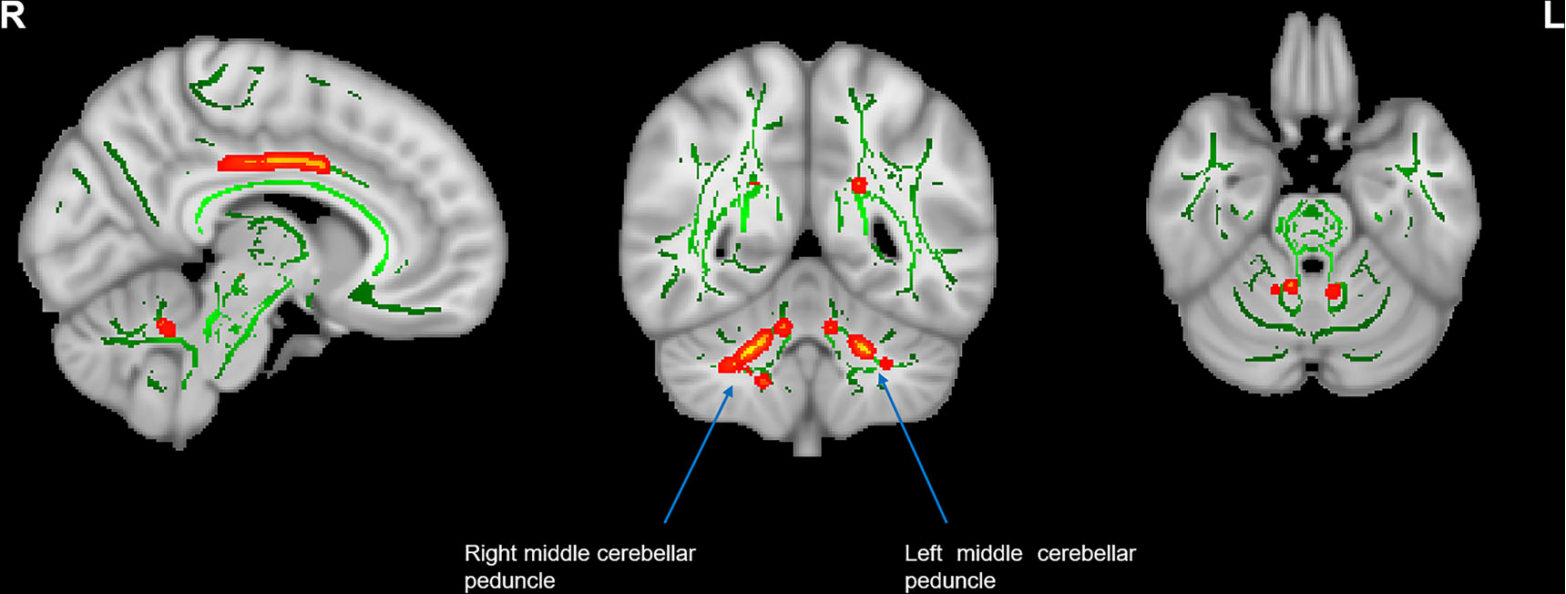
Brain white matter (WM) microstructural changes in EG patients compared with IG patients Decreased AD values in EG patients compared to IG patients were mainly found in the bilateral MCP (with FWE‐corrected *p* < .05). AD, axial diffusivity; EG, effective group; FWE, familywise error; IG, ineffective group; MCP, middle cerebellar peduncle

**FIGURE 4:**
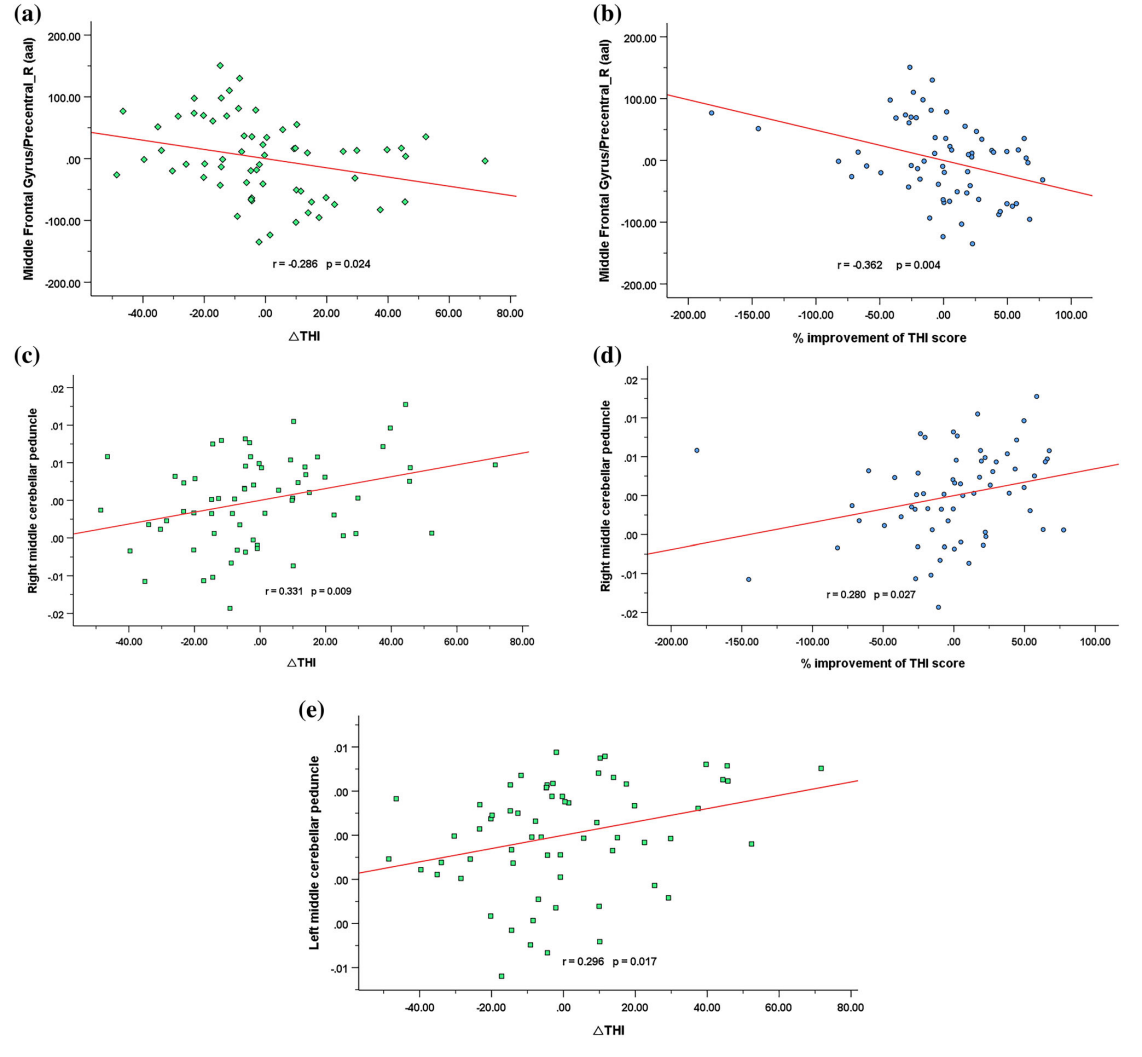
Correlations between brain structural and white matter (WM) microstructural changes and changes in THI scores and improvement of THI scores in tinnitus patients Pearson correlation showed a negative association between the GMV of the (a) right MFG/PreCG and ^Δ^THI scores (*r* = −.286, *p* = .024; uncorrected) and a negative association between the GMV of the (b) right MFG/PreCG and the % improvement of THI scores (r = −.362, *p* = .004; uncorrected). Pearson correlation showed a positive association between the AD value of the (c) right MCP and ^Δ^THI scores (*r* = .331, *p* = .009; uncorrected) and a positive association between the AD value of (d) the right MCP and the % improvement of THI scores (*r* = .280, *p* = .027; uncorrected). Pearson correlation showed a positive association between the AD value of the (e) left MCP and ^Δ^THI scores (*r* = .296, *p* = .017; uncorrected). AD, axial diffusivity; GMV, gray matter volume; MCP, middle cerebellar peduncle; MFG, middle frontal gyrus; PreCG, precentral gyrus; THI, Tinnitus Handicap Inventory

The three groups differed in WM indices in some auditory‐related and nonauditory‐related regions. Compared with the HC group, both the EG and IG showed decreased FA in the genu and splenium of the corpus callosum (CC), cingulum (cingulate gyrus), right inferior longitudinal fasciculus and inferior fronto‐occipital fasciculus, right superior longitudinal fasciculus and right superior temporal gyrus (STG) and increased MD in the body and genu of the CC and bilateral brainstem (Figure 2a, Table 3). Additionally, all tinnitus patients showed decreased AD in the cingulum (cingulate gyrus), bilateral middle cerebellar peduncle (MCP), left thalamus, left retrolenticular part of the internal capsule and right superior longitudinal fasciculus and increased RD in the genu and splenium of CC, cingulum (cingulate gyrus), right inferior longitudinal fasciculus and inferior fronto‐occipital fasciculus (Figure 2b, Table 3). When comparing the two groups of patients directly, the EG showed reduced AD in the bilateral MCP (Figure 3, Table 3). In addition, the AD value in the right MCP was positively correlated with the ^Δ^THI score and the % improvement of THI score in all tinnitus patients (*r* = .331, *p* = .009 and *r* = .280, *p* = .027, respectively) (Figure 4c,d), whereas the AD value in the left MCP was positively correlated with the ^Δ^THI score in all tinnitus patients (*r* = .296, *p* = .017) (Figure 4e).

**TABLE 3:** Difference in four white matter indices among EG, IG, and HCs according to the TBSS analysis (FWE voxel *p* < .05)

FA					
Anatomical region	MNI coordinate			Voxel size	Peak *p* value
	x	y	z		
EG/IG < HCs					
Genu of corpus callosum	2	21	15	2,149	.0002
Cingulum (cingulate gyrus) L	−10	−33	32	275	.0002
Cingulum (cingulate gyrus) R	9	17	29	235	.0002
Inferior longitudinal fasciculus and inferior fronto‐occipital fasciculus R	39	−9	−16	130	.0002
Superior longitudinal fasciculus R	46	−12	27	113	.0002
Splenium of corpus callosum	−14	−34	26	40	.0006
Right superior temporal gyrus	50	−34	14	34	.0018

MD					
Anatomical region	MNI coordinate			Voxel size	Peak *p* value
	x	y	z		
EG/IG > HCs					
Body of corpus callosum	4	12	21	143	.0006
Body of corpus callosum	−4	1	25	85	.0004
Body of corpus callosum	−13	−5	32	85	.0002
Genu of corpus callosum	−6	26	12	41	.0004
Right brainstem	7	−24	−13	39	.0002
Left brainstem	−4	−26	−13	24	.0006
Genu of corpus callosum	6	20	17	16	.0026

AD					
Anatomical region	MNI coordinate			Voxel size	Peak *p* value
	x	y	z		
EG/IG < HCs					
Cingulum (cingulate gyrus) L	−10	−31	32	145	.0002
Cingulum (cingulate gyrus) R	8	5	34	128	.0002
Right middle cerebellar peduncle	24	−51	−35	125	.0002
Left thalamus	−20	−25	−2	70	.0002
Left middle cerebellar peduncle	−21	−52	−33	62	.0024
Left retrolenticular part of internal capsule	−30	−26	4	36	.001
Right middle cerebellar peduncle	27	−48	−38	26	.0002
Superior longitudinal fasciculus R	41	−9	28	21	.0024
EG < IG					
Right middle cerebellar peduncle	24	−51	−35	125	.0002
Left middle cerebellar peduncle	−21	−52	−33	62	.0024

RD					
Anatomical region	MNI coordinate			Voxel size	Peak *p* value
	x	y	z		
EG/IG > HCs					
Genu of corpus callosum	−7	23	16	1,627	.0002
Cingulum (cingulate gyrus) L	−7	−12	34	172	.0002
Cingulum (cingulate gyrus) R	8	14	30	99	.0002
Inferior longitudinal fasciculus R	47	−1	−18	24	.0002
Inferior longitudinal fasciculus and inferior fronto‐occipital fasciculus R	39	−11	−14	16	.015
Splenium of corpus callosum	−13	−32	27	13	.0034

Abbreviations: AD, axial diffusivity; EG, effective group, FA, fractional anisotropy; FWE, family wise error; IG, ineffective group; HCs, healthy controls; MD, mean diffusivity; MNI, Montreal Neurological Institute; RD, radial diffusivity; TBSS, tract‐based spatial statistics.

### 3.4 The VBM change of the MFG/PreCG and the diffusion index alteration of bilateral MCP as indicators

**FIGURE 5:**
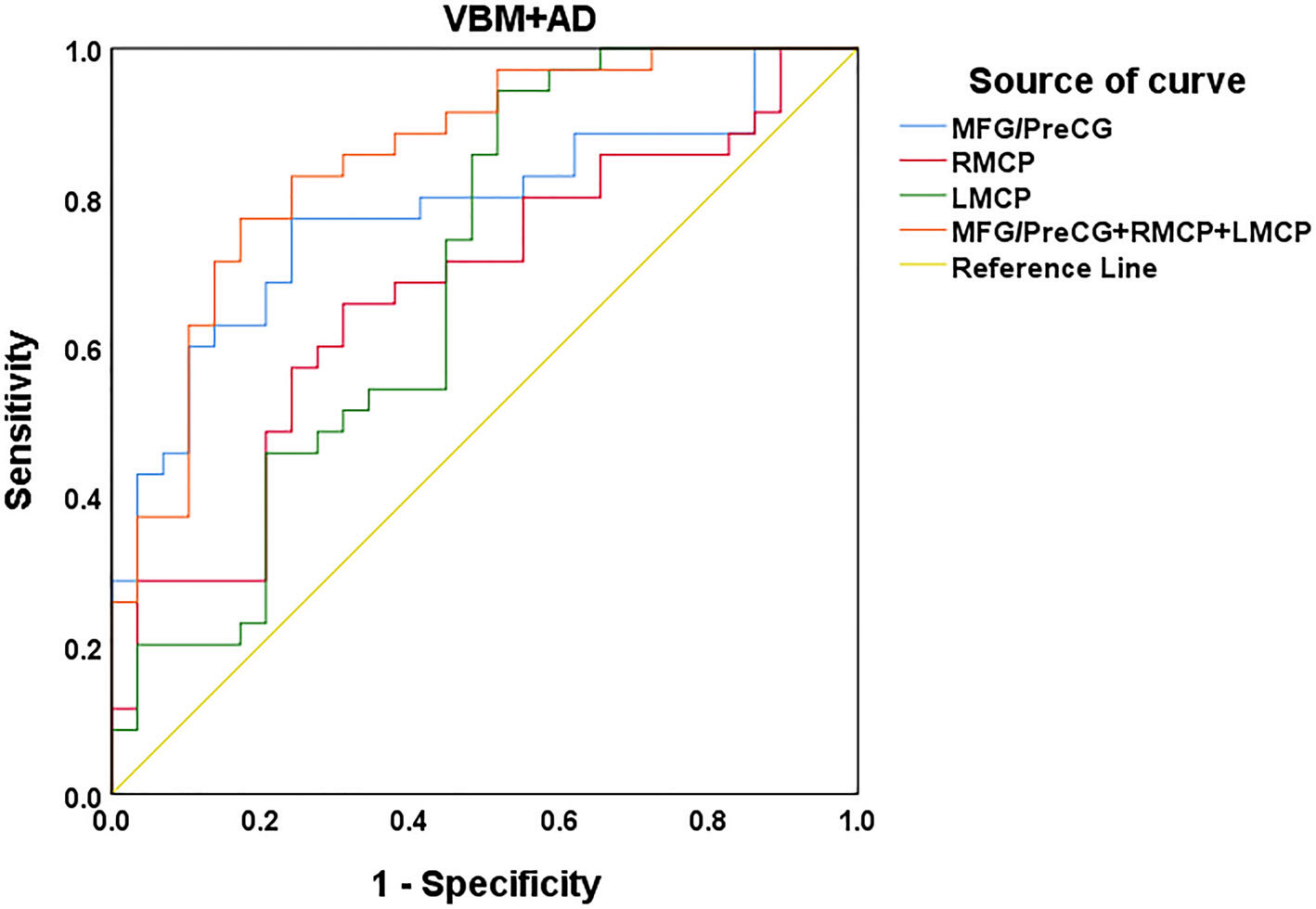
Receiver operating characteristic (ROC) curve for uncorrected VBM changes and diffusion index alterations as indicators An optimal diffusion metric cutoff value of the VBM changes in the MFG/PreCG and AD values in the bilateral MCP and their combination was determined at a sensitivity of 77.1, 65.7, 94.3, and 77.1% and a specificity of 75.9, 69.0, 48.3, and 82.8%, respectively. The areas under the curve (AUCs) for the ROC curve were 0.780, 0.673, 0.693, and 0.851 (95% confidence intervals from 0.666 to 0.895, 0.540 to 0.806, 0.557 to 0.828, and 0.757 to 0.945, respectively). The PPV and NPV for the gray matter change of the MFG/PreCG and the white matter (WM) integrity change in the bilateral MCP and the combination of them were 72.6 and 80.0%, 63.7 and 70.8%, 60.2 and 91.1%, and 78.8 and 81.9% separately. AD, axial diffusivity; MCP, middle cerebellar peduncle; MFG, middle frontal gyrus; PreCG, precentral gyrus

Figure 5 and Table 4 show the sensitivity and specificity of the gray matter change of the MFG/PreCG and the diffusion index alteration of the bilateral MCP in the tinnitus group for prognosis evaluation and screening patients before the sound treatment. Using the cutoff values of 0.530, 0.347, 0.426, and 0.599, we evaluated prognosis and screened patients with a sensitivity of 77.1% and specificity of 75.9% using the gray matter change of the MFG/PreCG, a sensitivity of 65.7% and specificity of 69.0% using the WM integrity of the right MCP, a sensitivity of 94.3% and specificity of 48.3% using the WM integrity of the left MCP, and a sensitivity of 77.1% and specificity of 82.8% using the combination of the gray matter change in the MFG/PreCG and the WM integrity change in the bilateral MCP separately. The area under curve (AUC) for the receiver operating characteristic curve values were 0.780 (95% confidence intervals from 0.666 to 0.895), 0.673 (95% confidence intervals from 0.540 to 0.806), 0.693 (95% confidence intervals from 0.557 to 0.828) and 0.851 (95% confidence intervals from 0.757 to 0.945), respectively. Moreover, the positive (PPV) and negative predictive value (NPV) for the gray matter change of the MFG/PreCG and the WM integrity change in the bilateral MCP and the combination of them were 72.6 and 80.0%, 63.7 and 70.8%, 60.2 and 91.1%, and 78.8 and 81.9%, separately.

**TABLE 4:** Results of ROC curve analysis of the brain structural and white matter microstructural reorganization as prognostic indicators

Brain reorganization	AUC	Sensitivity (%)	Specificity (%)	*p*‐Value	Cutoff	PPV (%)	NPV (%)
MFG/PreCG	0.780	77.1	75.9	.000	0.530	72.6	80.0
RMCP	0.673	65.7	69.0	.018	0.347	63.7	70.8
LMCP	0.693	94.3	48.3	.008	0.426	60.2	91.1
MFG/PreCG + RMCP + LMCP	0.851	77.1	82.8	.000	0.599	78.8	81.9

Abbreviations: AUC, area under curve; LMCP, left middle cerebellar peduncle; MFG, middle frontal gyrus; NPV, negative predictive value; PPV, positive predictive value; PreCG, precentral gyrus; RMCP, right middle cerebellar peduncle; ROC, receiver operator characteristic.

## 4 DISCUSSION

It is often seen in clinical settings that idiopathic tinnitus patients with similar THI scores may have different outcomes regarding the improvement after sound therapy; some patients show significant improvement of disease symptoms, whereas others show no obvious recovery or their symptoms may even continue to worsen. The present investigation addressed the question of whether different tinnitus symptom improvement outcomes can be attributed to differences in brain reorganization. Our findings indicated that brain structural and WM microstructural reorganization directly affected tinnitus outcomes at 6 months after sound therapy. Specifically, at the structural reorganization level, the IG showed more brain alterations in the MFG and M1 than the EG. The extent of the structural change was negatively associated with the ^Δ^THI score and % improvement of the THI score in all the patients with idiopathic tinnitus. At the WM microstructural reorganization level, the EG had significantly decreased AD in the bilateral MCP compared with the IG directly. The decreased AD was positively correlated with the ^Δ^THI score and/or % improvement of THI scores in all tinnitus patients. Our findings may shed some light on the role of brain reorganization during the recovery of tinnitus patients, may reveal indicators that can be used for the screening of patients and for making prognosis predictions before sound therapy, and further our understanding of the mechanisms underlying the recovery of tinnitus patients.

### 4.1 Brain structural alterations among the EG, IG, and HC group

Many studies have suggested that tinnitus can result in significant brain structural changes (gray matter atrophy) (Allan et al., 2016; Husain et al., 2011; Krick et al., 2015; Meyer et al., 2016; Schmidt et al., 2018), and the alterations occurred not only in auditory‐related brain regions but also in nonauditory‐related areas. In line with prior studies, we found that patients with idiopathic tinnitus displayed significant gray matter atrophy in some areas that are not directly related to auditory function compared with HCs. Furthermore, our study showed that the IG had more gray matter atrophy than the EG in the right MFG and PreCG. According to some previous studies (Husain et al., 2011; Xu et al., 2019), as part of the executive control network (ECN), the MFG is associated with cognitive function, attention, and emotion underlying tinnitus. Moreover, some functional and metabolic studies (Micarelli et al., 2017; Song, de Ridder, van de Heyning, & Vanneste, 2012; Xu et al., 2019) have suggested that changes in the MFG (the ECN region) were related to patients' memory retrieval function, unexpected auditory input impairment, and/or an emotional response to tinnitus‐related hearing loss or the involvement of cognitive control. It is widely known that the PreCG is the primary motor cortex, and evidence has indicated that it plays a role in auditory‐related functions. For example, Xu et al. (2019) believed that the PreCG might participate in word recognition and phonological processing. In addition, in the study of Feng et al. (2018), they found significant functional changes between the PreCG and some other regions in tinnitus patients, and they speculated that the changes may suggest that tinnitus was caused or modulated by signals from the somatosensory, somatomotor, and visual‐motor systems in some patients. Furthermore, we found that the gray matter change in the MFG/PreCG was negatively correlated with the changes and % improvement of THI scores. As the primary evaluation standard of tinnitus severity, the improvement of THI scores indicates that tinnitus patients' symptoms have improved significantly after sound therapy. Thus, in our study, the correlations between the structural changes and THI improvement in IG means that, compared to the EG, for the patients with poor treatment outcomes, the more obvious their brain alteration is, and the worse their prognosis is.

Taken together, we speculated that the structural changes in the MFG and PreCG may indicate that tinnitus is a consequence of the cross‐modal neural interaction of brain networks and that advanced cognitive brain areas play an important role (Feng et al., 2018; Xu et al., 2019). The structural remodeling of these two brain regions may indicate that the therapeutic effect of tinnitus patients is imperfect after sound therapy and that patients with these changes may not be suitable for treatment.

### 4.2 Brain WM microstructural alterations among the EG, IG, and HC group

Previous studies have demonstrated that tinnitus or tinnitus‐related hearing loss can result in significant alterations in WM integrity (Chen et al., 2020; Ryu et al., 2016; Schmidt et al., 2018; Seydell‐Greenwald, Raven, Leaver, Turesky, & Rauschecker, 2014). Apart from brain structural changes, in the present study, we also found brain WM microstructural changes in auditory‐related and nonauditory‐related regions in tinnitus patients compared with HCs. For example, we found significantly decreased FA and increased MD and RD in the main part (body, genu, and splenium) of the CC WM decreased FA and AD and increased RD in the cingulum WM, and decreased FA and AD in the superior longitudinal fasciculus WM. These changes, we infer, were consistent with our prior study (Chen et al., 2020). Meanwhile, tinnitus patients showed more serious and widespread WM integrity changes in this study. We observed brain WM integrity changes in the inferior longitudinal fasciculus and inferior fronto‐occipital fasciculus, STG, retrolenticular part of the internal capsule, thalamus, and brainstem. The STG is the main part of the primary auditory cortex (Mtui, Gruener, & Dockery, 2016), and the brainstem, as a relay station of auditory information transmission, plays an important role in generating and modulating tinnitus (Theodoroff & Kaltenbach, 2019). Moreover, WM fiber tracts in the thalamus, also called thalamic radiation, include acoustic radiation, which originates in the medial geniculate nucleus and terminates in the primary auditory cortex (Husain et al., 2011; Mtui et al., 2016). Thus, these changes indicated that tinnitus could cause direct damage to auditory‐related WM fibers. Although the inferior longitudinal fasciculus, inferior fronto‐occipital fasciculus, and retrolenticular part of the internal capsule are not directly related to auditory function, they do play an important role in the development of tinnitus and the resulting neurological dysfunction. As it connects the ipsilateral temporal and occipital lobes (Catani, Howard, Pajevic, & Jones, 2002), the main function of the inferior longitudinal fasciculus is to integrate information between the speech and auditory regions in the cortex. For the inferior fronto‐occipital fasciculus, which connects the ipsilateral frontal, posterior parietal and temporal lobes; connects ipsilateral frontal and occipital lobes; and interacts with the uncinate fasciculus (Catani et al., 2002), its main function is to integrate the auditory and visual association areas with the prefrontal cortex (Husain et al., 2011). In line with the study of Kim, Park, Kim, Lee, and Park (2009), the change in the diffusion index in the internal capsule may be caused by sensory deprivation or compensatory mechanisms that result from tinnitus, tinnitus‐related hearing loss or WM disordering caused by the expansion of other fibers into the region (Husain et al., 2011). Therefore, these findings suggest that tinnitus can also lead to significant impairment of WM integrity that is not directly related to auditory function.

One of the most interesting findings of our study was that significantly decreased AD values were observed in the bilateral MCP in the EG more than in the IG. In particular, the AD values in the MCP were positively correlated with an improvement of THI scores in all patients. As our previous study reported, AD is an indicator of axonal damage, is negatively correlated with axonal injury and positively correlated with axonal repair (Chen et al., 2020). For the change in AD value in the MCP, a previous case report proved that it is a large nerve bundle formed by afferent fibers from the contralateral pontine nuclei and that damage to it can cause tinnitus and hearing loss (Matsuda, Inagawa, & Amano, 1993). Moreover, Çavdar, Özgür, Kuvvet, Bay, and Aydogmus (2018) found in their study that the cerebellum connected the auditory cortices via the MCP, and Feng et al. (2018) demonstrated that the cerebellum plays an important role in the dysfunction caused by tinnitus. In addition, Shelton et al. (2017) reported that changes in the WM microstructure in the MCP are associated with executive function. Thus, we believed that the WM integrity change in the MCP in the EG compared with the IG indicated axonal injury caused by tinnitus. The correlation between it and the improvement of THI scores means that the more the WM microstructure in the MCP changed, the better the prognosis in tinnitus patients was. That is, patients with WM integrity changes in the MCP were more sensitive to sound therapy. Therefore, based on the information above, our study showed that tinnitus can result in significant reorganization in auditory‐related or nonauditory‐related WM integrity, and the changes in the MCP may indicate that the therapeutic effect of these tinnitus patients is perfect after sound therapy. These findings may be valuable indicators to use for patient screening before sound therapy and to assess treatment effects by monitoring dynamic brain reorganization during treatment.

### 4.3 The VBM change of the MFG/PreCG and the diffusion index alteration of bilateral MCP as indicators

In the current study, by using the combination of the changed GMV of the MFG and the PreCG and the altered WM integrity index of the bilateral MCP as a possible indicator, at the cutoff value of 0.599, we obtained a sensitivity of 77.1%, specificity of 82.8%, and AUC value of 0.851 and the PPV and NPV was 78.8 and 81.9% separately. Notably, this result shows that these factors could be used as valuable imaging indicators for idiopathic tinnitus patient screening and for making prognosis predictions before sound therapy.

To date, few studies have used brain alterations to screen patients before sound therapy and predict their prognosis. For example, a previous functional study used neural network nodes as indicators to predict improvement after sound therapy and found that the adjusted AUC values for the bilateral thalami were the highest, but both were under 0.8 (left, 0.745; right, 0.708) (Han, Na, et al., 2019; Han, Yawen, et al., 2019). In our study, we applied the combination of changes in gray matter and WM microstructure as an indicator to screen patients and evaluate the prognosis before treatment, which reached relative ideal specificity and sensitivity and PPV and NPV simultaneously.

### 4.4 Limitations

There are several limitations in our study that should be noted. First, in this study, although we obtained convincing results, the conclusion of this study still needs to be further verified using a larger sample size. Second, this study is a cross‐sectional study, and we will perform similar evaluations as a longitudinal study in the future to investigate the possible brain changes in the EG and IG before and after sound therapy. Third, we recruited only right‐handed subjects. In future studies, we will recruit left‐handed subjects as well. Fourth, a proportion of the patients in this study had different degrees of hearing loss. In future studies, we will recruit patients with tinnitus without hearing loss and patients with hearing loss without tinnitus to further understand the different therapeutic effects of tinnitus and hearing loss after sound therapy.

## 5 CONCLUSION

In conclusion, our study demonstrated that idiopathic tinnitus could cause significant brain structural and WM microstructural reorganization in auditory and nonauditory‐related regions. More interestingly, EG and IG patients showed distinct brain reorganization patterns. Structural atrophy of the MFG and PreCG was predictive of poor prognosis, whereas the more altered WM integrity of the bilateral MCP, the more tinnitus symptoms improved after sound therapy. Consequently, our results indicate that the reorganization of these brain areas or fiber bundles directly affects the improvement of tinnitus, and future sound therapy or related interventions should target them. The right MFG/PreCG and bilateral MCP may be indicators that can be used to predict prognoses in patients with idiopathic tinnitus and to screen patients before sound therapy.

## CONFLICT OF INTERESTS

The authors declare no conflict of interest.

## AUTHOR CONTRIBUTIONS

**Qian Chen** and **Han Lv**: Conducted the research project and conceived the study. **Qian Chen**: Wrote the manuscript. **Zhenchang Wang**, **Xuan Wei**, **Pengfei Zhao**, **Zhenghan Yang**, and **Shusheng Gong**: Provided technical and clinical support. **Zhenchang Wang**: Reviewed the manuscript and agree to be accountable for all aspects of the work.

## Data Availability

The datasets generated for this study are available on request to the corresponding author.
